# *In vitro* susceptibility testing of aztreonam-avibactam against predominantly NDM-producing *Enterobacterales* in Peru

**DOI:** 10.1128/spectrum.00762-26

**Published:** 2026-08-04

**Authors:** Coralith Garcia, Yelinda Reyes, Lizeth Astocondor, Liliana Caytuiro, Maribel Riveros, Catherine Amaro, Stephanie Andrade, Luis Hernández, Roxana Sandoval, Giancarlo Peréz-Lazo, Carolina Cucho, Manuel Melo, Ivan Sabogal, Liz Lino, Edwin Cuaresma, Araceli Olivares, Arnaldo Lachira, Kattya Michilot, Juan Ramos, Roberto Diaz, Alexander Briones, Segundo Burgos, Diego Cuicapuza, Pablo Tsukayama, Fiorella Krapp

**Affiliations:** 1 Instituto de Medicina Tropical Alexander von Humboldt, Universidad Peruana Cayetano Heredia 33216, Lima, Peru; 2 Hospital Nacional Cayetano Heredia 280152, Lima, Peru; 3 Hospital Nacional Guillermo Almenara Irigoyen EsSalud, Lima, Peru; 4 Hospital Nacional Dos de Mayo 504674, Lima, Peru; 5 Instituto de Investigaciones en Ciencias Biomédicas, Universidad Ricardo Palma 33224, Lima, Peru; 6 Hospital Nacional Daniel Alcides Carrión 269034, Callao, Peru; 7 Hospital III Daniel Carrión de EsSalud, Tacna, Peru; 8 Hospital Nacional Sergio E. Bernales, Lima, Peru; 9 Hospital III José Cayetano Heredia Red Asistencial EsSalud Piura, Piura, Peru; 10 Hospital Regional de Lambayeque, Lambayeque, Peru; 11 Hospital Regional de Loreto “Felipe Arriola Iglesias”, Iquitos, Peru; 12 Hospital Regional Pucallpa, Pucallpa, Peru; University of Guelph College of Biological Science, Guelph, Ontario, Canada

**Keywords:** antimicrobial susceptibility testing, NDM carbapenemase, *Enterobacterales*, aztreonam-avibactam

## Abstract

Metallo-β-lactamase-producing *Enterobacterales* are distributed worldwide, but some Latin American countries show a higher prevalence. Aztreonam-avibactam (ATM-AVI) may be an option for treating these infections. To evaluate *in vitro* susceptibility to aztreonam (ATM) alone and ATM-AVI in carbapenem-non-susceptible *Enterobacterales* isolates, based on the type of carbapenemase production, we prospectively collected carbapenem-non-susceptible *Enterobacterales* isolates from Peruvian hospitals during 2023–2024. Identification and susceptibility testing were performed by commercial panels and disk diffusion. Carbapenemases were detected by immunochromatography. ATM and ATM-AVI MICs were determined using broth microdilution panels with avibactam fixed at 4 µg/mL. The non-susceptible isolates to ATM-AVI and those with double production of carbapenemases underwent whole-genome sequencing. A total of 438 *Enterobacterales* isolates were analyzed; carbapenemase production was detected in 422 (96.3%) and NDM was the most frequent (61.9%). Coproduction of NDM + KPC in *K. pneumoniae* and NDM + OXA-48-like in *Escherichia coli* was observed. Overall, 99.3% were susceptible to ATM-AVI; MIC_50_ and MIC_90_ were 0.12 and 2 µg/mL, respectively. Overall, *K. pneumoniae* isolates had lower MIC_50_ and MIC_90_ values to ATM-AVI (0.12 and 0.5 µg/mL) compared to *E. coli* (0.5 and 4 µg/mL). Three *E. coli* isolates were resistant to ATM-AVI (MIC ≥ 8 µg/mL), they belonged to ST410, ST167, and ST10 and harbored a YRIN insertion in PBP3 along with CYM-type, PER-type, and CTX-M-type beta-lactamase genes. ATM-AVI demonstrated potent activity against carbapenem-non-susceptible *Enterobacterales*, including those producing NDM, which is the carbapenemase most frequently detected in Peruvian hospitals.

**IMPORTANCE:**

*Enterobacterales* isolates cause common illnesses in humans. Carbapenems are the antibiotics used to treat several of these infections, and increasingly, isolates resistant to these antibiotics are found. The most important mechanism of resistance to carbapenem among *Enterobacterales* is the production of enzymes called carbapenemases. Our results allowed us to recognize that NDM is the most frequent type of carbapenemase detected. Most of the antimicrobials available do not cover the *E*nterobacterales carrying NDM carbapenemase. In this scenario, we found that the new combination of drugs, aztreonam-avibactam, has high *in vitro* efficacy against most of the carbapenem-resistant isolates and against those isolates carrying NDM carbapenemase.

## INTRODUCTION

Carbapenem resistance among *Enterobacterales* species has been increasingly reported worldwide. The primary mechanism of this resistance is the production of carbapenemases, which are classified into three Ambler classes: A (KPC), B (NDM and other metallo-β-lactamases [MBLs]), and D (OXA-48-like). Each of these enzymes has presented distinct geographic and temporal distributions. Of special concern is the dissemination of NDM carbapenemase among *Enterobacterales* in Latin America (1–4).

Treatment options for carbapenem-resistant *Enterobacterales* (CRE) are limited worldwide but more critical in Latin America, where few last-generation antibiotics are available. Avibactam is a synthetic, non-β-lactam β-lactamase inhibitor with broad-spectrum activity against multiple β-lactamase classes, including class A (extended spectrum beta-lactamases [ESBLs] and KPC), class C (AmpC), and class D (OXA-48-like) (5). Additionally, avibactam has an elimination half-life of 2.5 h—longer than that of other β-lactamase inhibitors—which provides advantages in suppressing bacterial growth in infection models. Avibactam was approved in 2015 in combination with ceftazidime for the treatment of complicated urinary tract and intra-abdominal resistant infections (6). Ceftazidime-avibactam has been successfully used to treat infections caused by CRE (7); however, this combination lacks activity against isolates that produce MBLs such as NDM (8).

Although CRE isolates carrying MBLs should remain susceptible to aztreonam since MBLs cause hydrolysis of all beta-lactams except monobactams, these isolates often co-harbor ESBLs, which hydrolyze aztreonam and confer resistance (9). The combination of aztreonam and avibactam (ATM-AVI) should overcome this challenge, as avibactam inhibits ESBLs, thereby allowing aztreonam to regain activity against CRE. Karlowsky et al. evaluated 267 MBL-positive CRE isolates collected from multiple countries and observed a significant difference in the minimum inhibitory concentration (MIC_50_) values: from 64 µg/mL for aztreonam alone to 0.12 µg/mL for ATM-AVI (10). These findings highlight the *in vitro* efficacy of ATM-AVI against MBL-producing CRE isolates.

Observational studies provide evidence supporting the use of ceftazidime-avibactam plus aztreonam for infections caused by MBL-producing *Enterobacterales* isolates (5). On the other hand, the role of ATM-AVI has been analyzed in two clinical trials with a limited number of participants. However, the results showed a potential treatment option for these infections (11, 12).

The objective of this study was to assess the *in vitro* activity of ATM-AVI vs aztreonam alone against carbapenem-non-susceptible *Enterobacterales* isolates recovered in 10 Peruvian hospitals, and to evaluate if this activity varied based on the type of carbapenemase detected.

## MATERIALS AND METHODS

### Isolates collection

A laboratory network was established for surveillance from August 2023 to November 2024 to collect carbapenem-non-susceptible *Enterobacterales* isolates in 10 hospitals of Peru distributed in six regions. These isolates were recovered as part of routine patient care. Specimen sources included fluids, secretions, and blood (stool or rectal swabs were excluded). The isolates were transferred to Trypticase Soy Agar (TSA) tubes and sent periodically to the Instituto de Medicina Tropical Alexander von Humboldt where they were preserved for further testing. Isolates were excluded if they were identified as non-fermenting gram-negative bacilli, if they were duplicated, or if they were susceptible to the three carbapenems tested (ertapenem, imipenem, and meropenem). Only one isolate per person was further analyzed.

### Laboratory analysis

All isolates collected were retested. Identification and antimicrobial susceptibility testing to most antibiotics were performed using a commercial panel (Phoenix ID-406, Becton Dickinson, USA). Because amikacin was not included in this panel, the Kirby-Bauer method was used to test this antibiotic. Non-susceptibility to carbapenems was defined as intermediate susceptibility or resistance to any of the three carbapenems tested (meropenem, imipenem, or ertapenem). For MIC evaluation of ATM and ATM-AVI, broth microdilution was performed using the commercial panels Sensititre TM PER1AZTF (0.03–32 µg/mL) and EUAZAXLF (0.03/4–64/4 µg/mL) (Thermo Scientific, UK), respectively. A fixed concentration of 4 µg/mL of avibactam was used. Each isolate was analyzed three times; the mode was used to define the MIC value. Non-susceptibility to ATM-AVI was defined as intermediate susceptibility (8/4 µg/mL) or resistance (≥16/4 µg/mL) using CLSI 2026 breakpoints (13). Detection of carbapenemases was done by a rapid immunochromatographic method (NG-TEST CARBA-5, Biotech, France) which detects KPC, NDM, VIM, IMP, and OXA-48-like following the manufacturer’s instructions.

Detection of extended-spectrum β-lactamase (ESBL) production was done using a commercial panel (Phoenix ID-406, Becton Dickinson). The isolates without ESBL results by this panel were analyzed using the double-disc synergy method proposed by Jarlier et al. (14). ATCC *K. pneumoniae* 700603 and *Escherichia coli* 25922 were used as positive and negative controls, respectively, for ESBL detection.

Two groups of isolates underwent whole-genome long-read sequencing: (i) isolates non-susceptible to ATM-AVI and (ii) isolates with double production of carbapenemases. After reactivation of isolates in tryptic soy broth (TSB), genomic DNA was extracted using the ZymoBIOMICS DNA Microprep Kit (Zymo Research, USA). Libraries were prepared using the Rapid PCR Barcoding Kit and sequenced on an R10.4.1 flow cell with a MinION Mk1B device (Oxford Nanopore Technologies). Long reads were filtered to retain those with a minimum average quality score of 9 and a minimum length of 1,000 bp using Filtlong v0.2.1. Adapter sequences were removed with porechop_abi v0.5.0. *De novo* assembly was performed with Flye v2.9.6-b1802 using the high-quality reads model (--nano-hq). Plassembler v1.6.2 was applied to identify potential plasmid replicons from both the filtered reads and the assembled contigs. Chromosomal and plasmid contigs were then reoriented with Dnaapler v1.2.0.

Genome quality control was performed with CheckM v1.2.4 (https://github.com/Ecogenomics/CheckM), retaining only assemblies with completeness ≥95% and contamination ≤5%. Taxonomic assignment was verified using GTDB-Tk v2.5.2 (https://github.com/Ecogenomics/GTDBTk). Sequence types (STs) were determined according to the ecoli_achtman_4 scheme using MLST v2.23.0 (https://github.com/tseemann/mlst) and Kleborate (https://github.com/klebgenomics/Kleborate). Antimicrobial resistance (AMR) genes were detected with AMRFinderPlus v4.0.23 (coverage ≥90%, nucleotide identity ≥90%, database v2025-06-03.1). *E. coli* phylogroups were determined *in silico* using the ClermonTyping v23.06 algorithm (http://clermontyping.iame-research.center/). Plasmid replicons were detected with PlasmidFinder v2.1 (https://www.genomicepidemiology.org/). Genome annotation was performed with Prokka v1.14.6.

## RESULTS

A total of 655 isolates were collected from 10 sentinel hospitals. Of these, 121 were susceptible to carbapenems, 62 were duplicated isolates, 19 were identified as non-fermenting gram-negative bacilli, and 15 showed no growth, leaving 438 isolates for the analysis. Specimen sources were urine (*n* = 211, 48.2%), secretions (*n* = 119, 27.1%), blood (*n* = 52, 11.9%), and others (*n* = 56, 12.8%). The most common species detected were *K. pneumoniae* (*n* = 286, 65.3%), followed by *E. coli* (*n* = 103, 23.5%). The remaining 49 isolates (11.2%) were distributed among nine other species (Table 1).

**TABLE 1:** Distribution of carbapenemase types among carbapenem-non-susceptible *Enterobacterales* species collected at Peruvian hospitals between 2023 and 2024

Microorganism	Total, *n*	Carbapenemase type						None^a^ *n* (%)
		NDM *n* (%)	KPC *n* (%)	OXA-48-like *n* (%)	IMP *n* (%)	OXA-48-like + NDM *n* (%)	KPC + NDM *n* (%)	
*Klebsiella pneumoniae*	286	177 (61.9)	82 (28.7)	8 (2.8)	5 (1.7)	0	5 (1.7)	9 (3.2)
*Escherichia coli*	103	66 (64.1)	3 (2.9)	23 (22.3)	0	11 (10.7)	0	0
*Enterobacter cloacae*	15	11 (73.4)	2 (13.3)	0	0	0	0	2 (13.3)
*Citrobacter freundii*	11	1 (9.1)	10 (90.9)	0	0	0	0	0
*Proteus mirabilis*	6	2 (33.3)	0	0	0	0	0	4 (66.7)
*Klebsiella oxytoca*	4	3 (75.0)	1 (25.0)	0	0	0	0	0
*Klebsiella aerogenes*	4	4 (100.0)	0	0	0	0	0	0
*Providencia rettgeri*	3	3 (100.0)	0	0	0	0	0	0
*Morganella morganii*	3	2 (66.7)	0	0	0	0	0	1 (33.3)
*Providencia stuartii*	2	2 (100.0)	0	0	0	0	0	0
*Serratia fonticola*	1	0	1 (100.0)	0	0	0	0	0
Total	438	271 (61.9)	99 (22.6)	31 (7.1)	5 (1.1)	11 (2.5)	5 (1.1)	16 (3.7)

^a^ None indicates absence of detectable carbapenemases by the NG-TEST CARBA-5.

Overall, we found a low level of susceptibility to most of the antimicrobials tested except for ATM-AVI (99.3%) and tigecycline (79.5%) (Table 2). Tigecycline showed higher percentages of susceptibility rates among *E. coli* (100%) compared to *K. pneumoniae* (72%). Production of ESBL was found in 91.8% of isolates (Table 2).

**TABLE 2:** Antimicrobial susceptibility and extended-spectrum-β-lactamase (ESBL) production among carbapenem-non-susceptible *Enterobacterales* (all), *Klebsiella pneumoniae*, *Escherichia coli*, and other species^*h*^^,^^*i*^

	All species (*n* = 438)				*Klebsiella pneumoniae* (*n* = 286)				*Escherichia coli* (*n* = 103)				Other species (*n* = 49)			
	MIC (µg/mL)			% S	MIC (µg/mL)			% S	MIC (µg/mL)			% S	MIC (µg/mL)			% S
Antibiotic	50	90	Range		50	90	Range		50	90	Range		50	90	Range	
Ampicillin	>16	>16	≤4 to >16	0.2	>16	>16	>16	0.0	>16	>16	>16	0.0	>16	>16	≤4 to >16	2.0
Ampicillin sulbactam	>16/8	>16/8	8/4 to >16/8	0.2	>16/8	>16/8	16/8 to >16/8	0.0	>16/8	>16/8	>16/8	0.0	>16/8	>16/8	8/4 to >16/8	2.0
Amikacin^*a*^	–^*j*^	–	–	26.9	–	–	–	29.0	–	–	–	13.6	–	–	–	42.9
Aztreonam	>32	>32	≤0.03 to >32	8.4	>32	>32	0.06 to > 32	1.7	>32	>32	0.06 to >32	12.6	16	>32	≤0.03 to >32	38.8
Aztreonam-avibactam	0.12	2	≤0.03 to 32	99.3	0.12	0.5	≤0.03 to 4	100.0	0.5	4	≤0.03 to 32	97.1	0.06	1	≤0.03 to 4	100.0
Cefazolin^*b*^	>8	>8	8 to > 8	0.0	>8	>8	>8	0.0	>8	>8	8 to > 8	0.0	>8	>8	>8	0.0
Cefepime	>16	>16	≤1 to >16	3.2	>16	>16	≤1 to >16	0.7	>16	>16	≤1 to >16	4.9	>16	>16	≤1 to >16	14.3
Cefoxitin	>16	>16	≤4 to >16	5.7	>16	>16	≤4 to >16	4.6	>16	>16	≤4 to >16	9.7	>16	>16	≤4 to >16	4.1
Ceftazidime^*c*^	>16	>16	≤1 to >16	3.6	>16	>16	≤1 to >16	0.7	>16	>16	≤1 to >16	7.1	>16	>16	≤1 to >16	13.0
Ceftriaxone	>4	>4	≤1 to >4	2.1	>4	>4	≤1 to >4	0.7	>4	>4	≤1 to >4	4.9	>4	>4	≤1 to >4	4.1
Ciprofloxacin^*d*^	>2	>2	≤0.125 to >2	3.4	>2	>2	≤0.125 to >2	3.5	>2	>2	≤0.125 to >2	1.9	>2	>2	≤0.125 to >2	6.1
Ertapenem^*e*^	>1	>1	≤0.25 to >1	0.5	>1	>1	≤0.25 to >1	0.4	>1	>1	1 to > 1	0.0	>1	>1	≤0.25 to >1	2.0
Gentamicin	>8	>8	≤2 to >8	30.4	>8	>8	≤2 to >8	18.5	≤2	>8	≤2 to >8	61.2	>8	>8	≤2 to >8	34.7
Imipenem^*e*^	>8	>8	≤0.25 to >8	4.6	>8	>8	≤0.25 to >8	1.8	>8	>8	0.5 to >8	12.6	>8	>8	0.5 to >8	4.7
Meropenem^*e*^	16	>32	≤0.5 to >32	6.0	32	>32	≤0.5 to >32	2.1	16	>32	≤0.5 to >32	17.5	16	>32	≤0.5 to >32	4.2
Piperacillin tazobactam	>64/4	>64/4	≤4/4 to >64/4	1.8	>64/4	>64/4	≤4/4 to >64/4	1.8	>64/4	>64/4	≤4/4 to >64/4	0.0	>64/4	>64/4	≤4/4 to >64/4	6.1
Tigecycline^*f*^	2	4	≤1 to >4	79.5	2	4	≤1 to >4	72.0	≤1	2	≤1 to 2	100.0	2	4	≤1 to >4	86.1
TMP-SMX	>2/38	>2/38	≤0.5/9.5 to >2/38	13.9	>2/38	>2/38	≤0.5/9.5 to >2/38	15.0	>2/38	>2/38	≤0.5/9.5 to >2/38	9.7	>2/38	>2/38	≤0.5/9.5 to >2/38	16.3
ESBL production^*g*^, *n* (%)	402 (91.8)				283 (99.0)				96 (93.2)				23 (46.9)			

^*a*^ Amikacin susceptibility was tested by the Kirby-Bauer disk diffusion method.

^*b*^ Cefazolin MIC results were not obtained for 12 *Klebsiella pneumoniae*, 5 *Escherichia coli*, and 6 isolates from other species.

^*c*^ Ceftazidime MIC results were not obtained for 12 *Klebsiella pneumoniae*, 5 *Escherichia coli*, and 3 isolates from other species.

^*d*^ Ciprofloxacin MIC results were not obtained for one *Klebsiella pneumoniae* isolate.

^*e*^ For carbapenems, MIC results were not obtained for a small number of isolates: one *Klebsiella pneumoniae* for ertapenem, one *Klebsiella pneumoniae,* and six other species for imipenem, and one isolate from other species for meropenem. All these isolates are resistant or intermediate to meropenem.

^*f*^ Tigecycline MIC results were not obtained for 13 isolates from other species.

^*g*^ ESBL production was determined by microdilution (Phoenix ID-406, Becton Dickinson); isolates with indeterminate results were confirmed by the double-disk synergy test.

^*h*^ The MIC_50_, MIC_90_, and MIC range are presented along with the proportion of isolates susceptible to each antibiotic tested.

^*i*^ MIC, minimum inhibitory concentration; % S, percentage of susceptible isolate; TMP-SMX, trimethoprim sulfamethoxazole.

^*j*^ “−” indicates that MIC values were not calculated for amikacin.

Carbapenemase production was detected in 422 isolates (96.3%), in the remaining 16 isolates (3.7%) no carbapenemase was identified. The most frequently detected carbapenemase was NDM (*n* = 271, 61.9%), followed by KPC (*n* = 99, 22.6%), OXA-48-like (*n* = 31, 7.1%), and IMP (*n* = 5, 1.1%). Additionally, 16 isolates co-produced two carbapenemases: NDM + OXA-48-like (*n* = 11, 2.5%) and NDM + KPC (*n* = 5, 1.1%) (Table 1).

*K. pneumoniae* (*n* = 286) harbored all four detected carbapenemase types; the most frequent were NDM (*n* = 177, 61.9%) and KPC (*n* = 82, 28.7%). Only *K. pneumoniae* isolates showed coproduction of NDM + KPC (*n* = 5). Among *E. coli* isolates (*n* = 103), NDM (*n* = 66, 64.1%) was the predominant carbapenemase, followed by OXA-48-like (*n* = 23, 22.3%). All 11 co-producing NDM + OXA-48-like were *E. coli* (Table 1).

NDM was the most frequently detected carbapenemase in 9/10 hospitals located in 5/6 regions. Co-production of double carbapenemase was only observed in isolates recovered from three hospitals in Lima (capital of Peru) (Fig. S1).

Overall, only 8.4% of all isolates exhibited susceptible MICs to aztreonam while MIC_50_ and MIC_90_ were both 32 µg/mL. ATM-AVI MIC values ranged from ≤0.03 to 32 µg/mL, and the MIC_50_ and MIC_90_ were 0.12 and 2 µg/mL, respectively. Avibactam lowered the MIC_90_ of aztreonam for all isolates evaluated from >32 to 2 µg/mL. Overall, 99.3% of isolates were susceptible to ATM-AVI, while three (0.7%) *E. coli* isolates were non-susceptible, resulting in 97.1% susceptibility to ATM-AVI among *E. coli* isolates and 100% susceptibility among *K. pneumoniae* isolates (Table 2).

MIC_50_ for ATM-AVI was 0.25 µg/mL for isolates carrying KPC and 0.12 µg/mL for those carrying class B carbapenemase (mainly NDM) and OXA-48-like. MIC_90_ for ATM-AVI was 0.5, 1, and 2 µg/mL for KPC, class B carbapenemase, and OXA-48-like carrying isolates, respectively. *E. coli* producing NDM + ESBL had the highest MIC_50_ (0.5 µg/mL) and MIC_90_ values (4 µg/mL) compared to other mechanisms of resistance.

Overall, the isolates that co-produced two carbapenemases showed higher ATM-AVI MIC_50_ (2 µg/mL) and MIC_90_ (4 µg/mL) values compared to those that carried one carbapenemase. When carbapenemase co-producing isolates were compared, those carrying NDM + OXA-48-like showed higher ATM-AVI MIC_50_ (2 µg/mL) and MIC_90_ values (4 µg/mL) compared to those carrying NDM + KPC (0.25 µg/mL). Regardless of the type of carbapenemases, *K. pneumoniae* had lower MIC_50_ and MIC_90_ values compared to *E. coli*, 0.12 and 0.5 µg/mL compared to 0.5 and 4 µg/mL, respectively (Table 3).

**TABLE 3:** Distribution of MIC_50_ and MIC_90_ values for aztreonam and aztreonam-avibactam among carbapenem-non-susceptible *Enterobacterales* isolates by carbapenemase type and by species

	*n* (%)	Aztreonam			Aztreonam-avibactam		
		MIC (µg/mL)^*a*^			MIC (µg/mL)^*a*^		
		50	90	Range	50	90	Range
Carbapenemase type							
One carbapenemase	406 (92.7)	>32	>32	≤0.03 to >32	0.12	1	≤0.03 to 32
Class A	99 (22.6)	>32	>32	32 to >32	0.25	0.5	≤0.03 to 4
KPC + ESBL (*K. pneumoniae*)	82 (18.7)	>32	>32	32 to >32	0.25	0.5	0.06–4
KPC + ESBL (*E. coli*)	3 (0.7)	−	−	>32	−	−	≤0.03 to 0.12
KPC + ESBL (other species)	4 (0.9)	−	−	>32	−	−	0.06–0.25
KPC (other species)	10 (2.3)	>32	>32	32 to >32	0.12	0.25	0.06–1
Class B	276 (63.0)	>32	>32	≤0.03 to >32	0.12	1	≤0.03 to 32
NDM + ESBL (*K. pneumoniae*)	177 (40.4)	>32	>32	0.06 to >32	0.12	0.25	≤0.03 to 4
IMP + ESBL (*K. pneumoniae*)	4 (0.9)	−	−	>32	−	−	≤0.03 to 0.06
IMP (*K. pneumoniae*)	1 (0.2)	−	−	0.06	−	−	0.06
NDM + ESBL (*E. coli*)	65 (14.8)	>32	>32	0.12 to >32	0.5	4	≤0.03 to 32
NDM (*E. coli*)	1 (0.2)	−	−	4	−	−	4
NDM + ESBL (other species)	17 (3.9)	16	>32	0.12 to >32	0.06	2	≤0.03 to 4
NDM (other species)	11 (2.5)	0.12	0.25	≤0.03 to 0.25	0.06	0.12	≤0.03 to 0.25
Class D	31 (7.1)	>32	>32	0.06 to >32	0.12	2	≤0.03 to 4
OXA-48-like + ESBL (*K. pneumoniae*)	6 (1.4)	>32	>32	32 to >32	0.12	0.12	0.06–0.12
OXA-48-like (*K. pneumoniae*)	2 (0.5)	−	−	0.06–0.12	−	−	0.06
OXA-48-like + ESBL (*E. coli*)	17 (3.9)	>32	>32	8 to >32	2	2	≤0.03 to 4
OXA-48-like (*E. coli*)	6 (1.4)	0.12	4	0.06–4	0.06	2	≤0.03 to 2
Double carbapenemase	16 (3.7)	>32	>32	>32	2	4	≤0.03 to 4
NDM + KPC + ESBL (*K. pneumoniae*)	5 (1.1)	>32	>32	>32	0.25	0.25	0.12–0.25
NDM + OXA-48-like + ESBL (*E. coli*)	11 (2.5)	>32	>32	>32	2	4	≤0.03 to 4
None^*b*^	16 (3.7)	16	>32	≤0.03 to >32	0.12	2	≤0.03 to 2
Species							
*Klebsiella pneumoniae*	286 (65.3)	>32	>32	0.06 to >32	0.12	0.5	≤0.03 to 4
*Escherichia coli*	103 (23.5)	>32	>32	0.06 to >32	0.5	4	≤0.03 to 32
Other species	49 (11.2)	32	>32	≤0.03 to >32	0.06	1	≤0.03 to 4
All	438 (100)	>32	>32	≤0.03 to >32	0.12	2	≤0.03 to 32

^*a*^ “−” indicates that MIC_50_ and MIC_90_ were not calculated if the number of isolates was less than 5.

^*b*^ None indicates absence of detectable carbapenemases by the NG-TEST CARBA-5.

**Fig 1:**
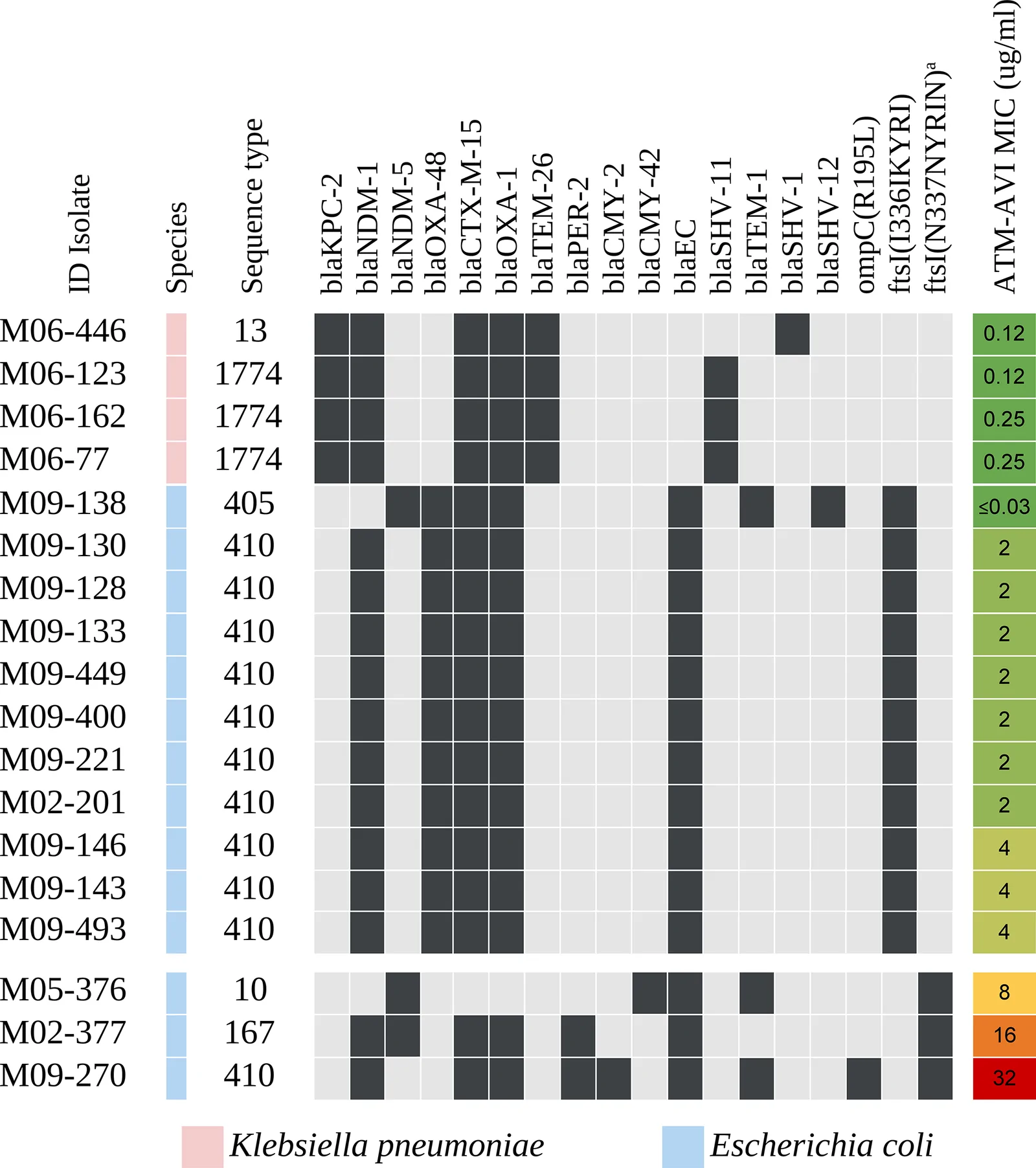
Genomic characteristics and susceptibility to aztreonam-avibactam of *Enterobacterales* isolates from Peruvian hospitals. ATM-AVI: Aztreonam-Avibactam; MIC, minimum inhibitory concentration. The insertion was identified and confirmed through variant calling using Medaka as *ftsI*(N337NYRIN), left-normalization of the indel, and alignment against reference wild-type ftsI sequence (*E. coli* K-12 MG16555, UniProt P0AD68) confirmed that it corresponds to the previously reported 333-YRIN insertion.

The three *E. coli* isolates non-susceptible to ATM-AVI were sequenced. All three carried a tetrapeptide insertion (YRIN) at position 333 in penicillin-binding protein 3 (PBP3). These strains belonged to ST410, ST167, and ST10, and they all carried MBL carbapenemase genes (NDM-5, NDM-1, or both) in addition to other AmpC variants (blaEC-15, blaCMY-42, and/or blaCMY-2), while two also carried CTX-M-15 and blaPER-2 genes (Fig. 1). Fifteen of the 16 isolates with double production of carbapenemases were sequenced, 4 *K. pneumoniae* and 11 *E. coli*. The four *K. pneumoniae* isolates carried carbapenemases NDM-1 and KPC-2; three belonged to ST1774 and one to ST16. Among the 11 *E. coli* isolates, 10 of them carried NDM-1 and OXA-48 carbapenemases and belonged to ST10. The remaining isolate carried NDM-5 and OXA-48 carbapenemases and belonged to ST405 (Fig. 1). All these *E. coli* isolates were susceptible to ATM-AVI and carried a different tetrapeptide insertion (IKIR) in PBP3 (Fig. 1).

## DISCUSSION

We found that *K. pneumoniae* and *E. coli* were the most frequent carbapenem-non-susceptible *Enterobacterales* isolates, with carbapenemases NDM, KPC, and OXA-48-like present in both species. Overall, NDM was the most frequently detected carbapenemase in 9/10 participating hospitals distributed in 5/6 Peruvian regions. These isolates were susceptible to ATM-AVI except for three *E. coli* isolates.

During the COVID-19 pandemic, several Latin American countries reported a higher frequency of CRE isolates, the emergence of new carbapenemases, and cases co-harboring two or more carbapenemases. In 2021, the co-production of NDM and KPC was first reported in Argentina, Costa Rica, Ecuador, Guatemala, Paraguay, Peru, and Venezuela. That same year, Chile reported its first detection of OXA-48 (15). A multicenter study in Peru between 2017 and 2019 analyzed blood isolates collected from 15 hospitals and found that 11% of *K. pneumoniae* isolates were resistant to carbapenems, whereas no *E. coli* isolates exhibited carbapenem resistance (16). In 2019, the Peruvian National Reference Laboratory analyzed isolates from 30 institutions across 12 regions and reported only *K. pneumoniae* and *E. coli* producing NDM or KPC carbapenemases (17). Our study performed during the post-COVID period identified NDM, KPC, OXA-48-like, and IMI carbapenemases distributed among 11 *Enterobacterales* species from several regions, as well as isolates carrying two carbapenemases. These findings raise the question of whether the COVID-19 pandemic had an effect on the epidemiology of carbapenemase-producing *Enterobacterales* in Peru.

The epidemiology of carbapenemase production varies across countries and regions. In the post-COVID-19 period, the detection of KPC carbapenemase was significantly higher than NDM among CRE isolates in Ecuador (86.7% vs 11.7%, respectively). In contrast, in Chile, the proportion of KPC-producing isolates was only slightly higher than that of NDM (46.0% vs 42.0%) (18, 19). In Argentina, an analysis of CRE isolates from 182 hospitals revealed that the proportion of NDM-producing isolates slightly exceeded that of KPC producers (41.4% vs 39.8%) (20). Additionally, OXA-48 was detected at low levels in Argentina and Ecuador but was absent in Chile. The production of dual carbapenemases was identified in Argentina and Chile but not in Ecuador, though it is important to note that the Ecuadorian study included a limited number of isolates (18–20). In a recent study that involved 490 MBL-producing *Enterobacterales* isolates from 22 countries, NDM-1 was the most frequently detected carbapenemase (21). Our findings align with this study since we found the predominance of NDM production in most hospitals. The molecular epidemiology of these infections remains a dynamic process. A 2015 study in a Peruvian hospital reported the first introduction of KPC-2-producing *K. pneumoniae*, which was later displaced by NDM-1-producing *K. pneumoniae*, becoming the predominant carbapenemase in the following years (22).

We found that the combination of ATM-AVI has potent activity against all carbapenemase-producing *Enterobacterales* isolates, including NDM producers. Comparable results have been reported previously. In 2021, Echegorry et al. analyzed a collection of isolates from Argentina and found that 96.3% of CRE isolates were susceptible to ATM-AVI (20). These *in vitro* results suggest that ATM-AVI is a potent antibiotic. The Infectious Diseases Society of America has suggested the use of ceftazidime-avibactam plus aztreonam for the treatment of MBL-producing *Enterobacterales* infections since ATM-AVI was not available yet in the United States when this guideline was released (23). To date, ATM-AVI is commercially available in some European countries, the United States, and Chile; however, it is not available in Peru yet.

Regarding the differences in ATM-AVI MIC values based on species, we found higher MIC_50_ and MIC_90_ values for *E. coli* compared to *K. pneumoniae*, similar to two studies that analyzed CRE isolates (mostly NDM-producers) from centers in the United States. In a first study, *E. coli* had higher MIC_50_ and MIC_90_ values than *K. pneumoniae* (4 and 8 µg/mL vs 0.25 and 1 µg/mL, respectively), and in a second study, a similar trend was observed (2 and 8 µg/mL vs 0.25 and 0.5 µg/mL, respectively) (24, 25). Additionally, all *K. pneumoniae* isolates were susceptible to ATM-AVI compared to 97.1% of *E. coli* isolates. This difference in susceptibility based on the species has also been seen in a study in the United Kingdom, where 100% of NDM-producing *K. pneumoniae* and *E. cloacae* isolates analyzed were susceptible to ATM-AVI, compared with 85.3% of NDM-producing *E. coli* (26).

Multiple studies have associated the reduced susceptibility to ATM-AVI with the insertion of four amino acids in PBP3 and the co-carriage of CMY-42 or other plasmid-mediated AmpC β-lactamases in *E. coli* strains (27–29). This phenomenon has also been reported epidemiologically among NDM-producing *E. coli* with PBP3 insertions and co-production of AmpC/ESBL in high-risk lineages such as ST410, ST167, and ST361, which are linked to broad β-lactam resistance (30, 31). In addition, carriage of PER-2 β-lactamases has been described in ATM-AVI-resistant strains (29), along with the demonstration of less efficient inhibition of PER-2 β-lactamase by avibactam, compared to other class A β-lactamases, supporting its role in decreased susceptibility to ATM-AVI (29, 32). In our three ATM-AVI non-susceptible NDM-producing *E. coli* isolates, we observed a stepwise MIC gradient to ATM–AVI: 8 µg/mL (PBP3–YRIN + CMY-42, PER-2 negative); 16 µg/mL (PBP3–YRIN + CTX M-15, PER-2, CMY-negative); and 32 µg/mL (PBP3–YRIN + CTX M-15 + PER-2 + CMY-2). These data indicate a progressive, additive effect whereby resistance to ATM–AVI is not driven solely by the MBL but rather by the convergence of PBP3 insertions and serine β-lactamases that together amplify the resistant phenotype (27, 33, 34). Two of the three isolates corresponded to sequence types previously associated with ATM-AVI resistance and PBP3 insertions (ST410 and ST167) (28). The remaining isolate belonged to ST10, which, to our knowledge, has not been previously reported but belongs to the same clonal complex (CC10) as ST167. These genes and PBP3 insertion were not observed in the 11 *E. coli* isolates susceptible to ATM-AVI that were also sequenced.

Our study has several limitations. First, one limitation was the lack of *in vitro* evaluation of other antibiotics that may be effective for the treatment of these multidrug-resistant infections, such as ceftazidime-avibactam, imipenem-relebactam, meropenem-vaborbactam, or colistin. Nevertheless, the predominance of MBL production may significantly limit the utility of some of these antibiotics. Second, while we were able to identify possible determinants of decreased susceptibility to ATM-AVI through WGS, we only sequenced a small proportion of *E. coli* isolates susceptible to ATM-AVI for comparison; therefore, we cannot assure that the identified mutations or genes are exclusive to non-susceptible isolates. Third, we did not sequence the isolates that were non-susceptible to carbapenems but did not produce carbapenemases, in order to recognize other non-enzymatic mechanisms of resistance.

In conclusion, NDM is the carbapenemase most frequently detected among *Enterobacterales* species in Peruvian hospitals and is mainly produced by *K. pneumoniae* and *E. coli* isolates. The combination of ATM-AVI has potent activity against carbapenem-non-susceptible isolates, including those producing NDM carbapenemase.

## Data Availability

The raw read files and assemblies for the 3 ATM-AVI non-susceptible *E. coli* isolates and the 15 dual-carbapenemase-producing *Enterobacterales* isolates have been deposited in GenBank at NCBI under accession numbers PRJNA1439406 and PRJNA1443524, respectively.

## SUPPLEMENTAL MATERIAL

Figure S1 spectrum.00762-26-s0001.docx Distribution of carbapenemases among 422 *Enterobacterales* isolates collected form 10 Peruvian collaborating hospitals.

ASM does not own the copyrights to Supplemental Material that may be linked to, or accessed through, an article. The authors have granted ASM a non-exclusive, world-wide license to publish the Supplemental Material files. Please contact the corresponding author directly for reuse.
