# Supplementary material for: In vitro susceptibility testing of aztreonam-avibactam against predominantly NDM-producing Enterobacterales in Peru
Source: Microbiol Spectr. 2026 Aug 4;14(9):e00762-26. doi: 10.1128/spectrum.00762-26 (PMC13532284; doi:10.1128/spectrum.00762-26)
Supplement: Supplementary file 1 [file spectrum.00762-26-s0001.docx]

**Supplemental Figure 1: Distribution of carbapenemases among 422 *Enterobacterales* isolates collected form 10 Peruvian collaborating hospitals.**
